# Supine Bridge Exercise for Low Back Pain: A Fascial Approach for Movement Impairment Syndromes (Part II)

**DOI:** 10.7759/cureus.83785

**Published:** 2025-05-09

**Authors:** Saverio Colonna, Antonio D'Alessandro, Riccardo Tarozzi, Fabio Casacci

**Affiliations:** 1 Rehabilitation Medicine, Spine Center, Bologna, ITA; 2 Research and Development, Osteopathic Spine Center Education (OSCE), Bologna, ITA; 3 Osteopathic Medicine, Osteopathic Spine Center Education (OSCE), Bologna, ITA; 4 Rehabilitation, Spine Center, Bologna, ITA

**Keywords:** fascial system, fascial tone, low back pain, lumbar stabilization, movement system impairment syndromes, supine bridge exercise, therapeutic intervention, trunk muscle activation

## Abstract

The supine bridge exercise (SBE) is widely used in rehabilitation for individuals with low back pain (LBP) due to its ability to enhance spinal stability and neuromuscular control. This second installment of a three-part review builds on previously discussed biomechanical principles to offer a functional rationale for applying the SBE to LBP subgroups identified through the Movement Impairment Syndromes framework. A novel interpretative model is proposed in which the SBE is reconceptualized not solely as a muscular strengthening tool, but as a method to increase passive fascial stiffness and promote segmental spinal stability. Emphasis is placed on the relationship between lumbar curvature, fascial tension, and motor control, with distinct execution strategies recommended for flexion-based and extension-based LBP. In flexion-related cases, the goal is to activate lumbar paraspinals while minimizing hamstring involvement; in extension-based presentations, limiting lumbar extension, engaging abdominal control, and promoting posterior pelvic tilt are prioritized. This article highlights the value of individualized, evidence-informed protocols that integrate fascial and biomechanical considerations to optimize rehabilitation outcomes for patients with LBP.

## Introduction and background

In recent years, the importance of core strength and stability has been widely recognized in the contexts of both rehabilitation and athletic training [1]. Core muscles serve as the center of the functional kinetic chain, contributing to resistance against spinal perturbations [2] and facilitating the transfer of power to distal segments during athletic activities [3]. In rehabilitation, core-strengthening exercises can reduce the risk of injury by enhancing both muscle strength and endurance [4]. Typical core-strengthening exercises include the supine bridge exercise (SBE), the prone bridge, and crunch exercises [5]. The bridge exercise is among the most studied and frequently used in rehabilitation. A Google Scholar search for “bridge exercise” AND “rehabilitation” yields approximately 1,400 results. This type of exercise is recommended for the prevention of hamstring injuries [6], iliopsoas tendinopathy [7], and chronic non-specific low back pain [8]. Furthermore, the literature describes several execution modalities, each indicating which muscle groups are primarily involved [9].

This article serves as a continuation of Part I of this work [9], which, from a biomechanical perspective, describes the main muscles activated during the classic SBE and its variations. The present work proposes an original rationale for the use of the SBE in the treatment of low back pain (LBP). Furthermore, it lays the groundwork for the upcoming article (Part III), which will present the use of SBE for the prevention and treatment of degenerative hip pathology.

## Review

Supine bridge exercise and LBP

Numerous research studies have provided evidence that LBP is associated with weakness and a lack of endurance in the lumbar extensor muscles [10-13]. Chronic non-specific LBP leads to dysfunction in trunk musculature; conversely, trunk muscle dysfunction may also contribute to the persistence of LBP [14-16]. Several studies have emphasized the benefits of supervised exercise in individuals with chronic LBP, although there is no clear evidence that one specific type of exercise is superior to others [17].

Additionally, some studies have shown that patients with LBP exhibit lower electromyographic activity in trunk muscles during bridging exercises (both supine and prone) compared to healthy adults [18]. For these reasons, the bridge exercise is frequently prescribed in rehabilitation programs targeting the lower limbs and the lumbopelvic region [19]. It also serves as a practical, reliable, and well-tolerated clinical tool for assessing lumbar spine stabilization endurance in individuals with LBP [18]. For instance, healthy individuals can maintain the supine bridge position with full hip extension for significantly longer durations (170 seconds) compared to those with chronic mechanical LBP (76 seconds) [18].

Before addressing the application of the SBE in pathological lumbar conditions, it is important to explore how therapeutic exercise can address dysfunctions that underlie lumbar pathology. To provide indications on how and when to utilize the SBE in lumbar disorders, a preliminary discussion is necessary to explore several key aspects, including: the relationship between lumbar curvature and LBP; importance of subgroup classification in patients with LBP; relevance of the fascial component in maintaining physiological spinal curves; spinal stability; the contribution of the lumbar myofascial system to LBP treatment; hamstrings and their relationship with LBP; tightness of the posterior lumbar muscles and LBP; the role of exercise in fascial tightness; application of the SBE in treating the lumbar myofascial component of LBP.

Relationship between lumbar curvature and LBP

The spine physiologically exhibits curvature in the sagittal plane, characterized by cervical and lumbar lordosis and thoracic kyphosis. Among spine specialists, the lumbar lordotic curvature (LLC) is frequently considered a potential predictive factor for LBP. A widely held view is that the LLC itself, or more specifically, its exaggeration, may be a contributing factor to LBP [20-22]. According to Cailliet [22], 75% of static and postural back pain may be associated with increased lumbar lordosis resulting from a greater lumbosacral angle. As a result, it is commonly believed that any physical activity promoting increased lordosis should be avoided [23].

However, several studies [24-26] have reported that loss of lordosis, not exaggeration, may be a more reliable predictor of LBP. As early as 1975, Magora [27], in a study involving subjects with and without LBP, reported that hyperlordosis was a common postural condition among healthy individuals, thereby limiting its value as a predictor of LBP. In contrast, intense pain appeared more frequently in individuals with hypolordosis. In Magora’s study, 5.8% of the 429 individuals in the LBP group had a flat back, whereas none of the 271 controls exhibited this feature. Magora [27] further emphasized that the loss of lordosis was particularly evident in occupations associated with herniated discs or severe low back pain. Hypolordosis may also occur as a result of poor posture or pregnancy and is generally difficult to maintain voluntarily in an upright position unless caused by a local pathological process structurally linked to the spine. The findings of a systematic review and meta-analysis [25] indicate a strong correlation between LBP and reduced lumbar lordosis, particularly when compared with age-matched healthy controls. Specifically, conditions such as lumbar disc herniation or disc degeneration were shown to be significantly associated with decreased LLC [28,29].

It is worth noting that a previous systematic review and meta-analysis [30] on this topic concluded that LLC does not differ significantly between individuals with and without LBP. Conversely, another study [31] found a correlation between increased lumbar curvature and LBP. These conflicting findings underscore the complexity of spinal alignment’s relationship with LBP. Nonetheless, the loss of LLC is a distinctive finding in the aging lumbar spine [32], and the prevalence of LBP increases with age [33]. Therefore, the LLC-LBP relationship has substantial clinical implications and serves as a foundational concept in functionally based treatment and prevention approaches for LBP.

Importance of subgroup classification in patients with LBP

The contradictory findings described above may be explained by the generic use of the term “low back pain” in the literature. This diagnostic label is primarily based on the patient's subjective perception of pain in the lumbar region. While this pain-based definition is useful for identifying a condition broadly referred to as “low back pain,” it would be inaccurate to assume that a single underlying functional cause is responsible for all cases.

In fact, the etiopathogenetic mechanisms underlying LBP may vary considerably and, as such, require different intervention strategies. Therefore, when referring to this condition, it would be more appropriate to use the plural form “low back pains” rather than the singular “low back pain.” This diagnostic imprecision may explain why some studies [34], after analyzing the relationship between lumbar curvature and chronic LBP, concluded that radiographic assessments were unnecessary, claiming no correlation between lumbar curvature and pain.

According to Van Dillen et al. [35-37], patients with non-specific LBP should be classified into homogeneous subgroups based on consistent symptom-provoking patterns and standardized clinical tests (provocation/relief tests) that apply various types of mechanical loads (flexion, extension, rotation) to the lumbar spine. This classification model is thoroughly discussed in Sahrmann’s text, Diagnosis and Treatment of Movement Impairment Syndromes [38]. In this framework, subgroups are named according to the directions of movement and postures that consistently exacerbate the patient’s symptoms (provocation tests) and that improve when these postures and movements are systematically modified to alter the specific load applied to the lumbar spine (relief tests).

**Figure 1:**
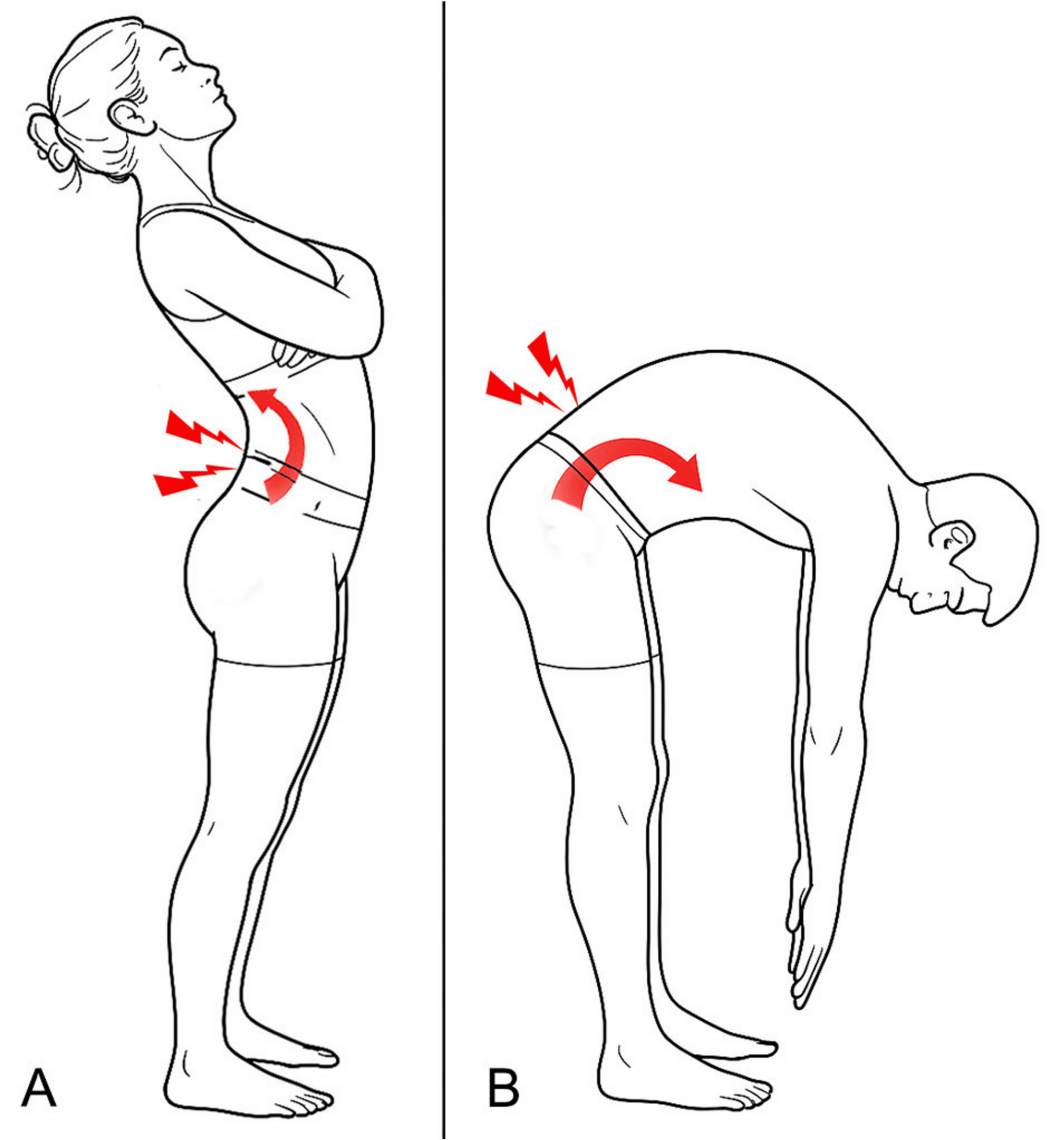
Illustration of fundamental movement patterns outlined in the Movement Impairment Syndromes framework A: Extension-based movement impairment syndromes; B: Flexion-based movement impairment syndromes Image credit: Author Saverio Colonna

It has been reported [39] that patients classified under the lumbar extension-rotation subgroup tend to stand with greater lumbar lordosis compared to patients in other LBP subgroups and asymptomatic individuals. These patients report increased symptoms during clinical tests involving extension (Figure 1A) or rotation of the lumbar spine [40] and a reduction in symptoms when the extension or rotation load is diminished [35,41]. Therefore, standing posture tends to provoke more discomfort in individuals within the extension or extension-rotation subgroups compared to other LBP classifications. This may be due to increased mechanical loading on the posterior spinal structures during prolonged standing. In these patients, lumbar lordosis is generally more pronounced than in other LBP subgroups and asymptomatic individuals [31]. In contrast, patients classified under the flexion (Figure 1B) and/or flexion-rotation syndromes tend to present with a flattened lumbar spine. This group commonly reports pain during or after adopting flexed postures, such as bending forward or prolonged sitting.

In such patients, strategies aimed at increasing the tightness of posterior fascial systems, such as the sacrotuberous and sacrospinous ligaments, thoracolumbar fascia, and the aponeuroses of the erector spinae and multifidus, may provide therapeutic benefit [42,43]. Sadeghisani et al. [44] observed that patients with flexion-rotation syndrome exhibited greater and earlier pelvic rotation in the transverse plane during prone hip internal rotation testing, along with reduced hip internal rotation range (though the latter was not statistically significant). Having introduced the importance of functional classification in LBP, we can now explore the rationale behind using the SBE in various clinical presentations, supported by the extensive literature on bridging and its modifications [45-47].

The importance of the fascial component in maintaining physiological spinal curves

**Figure 2:**
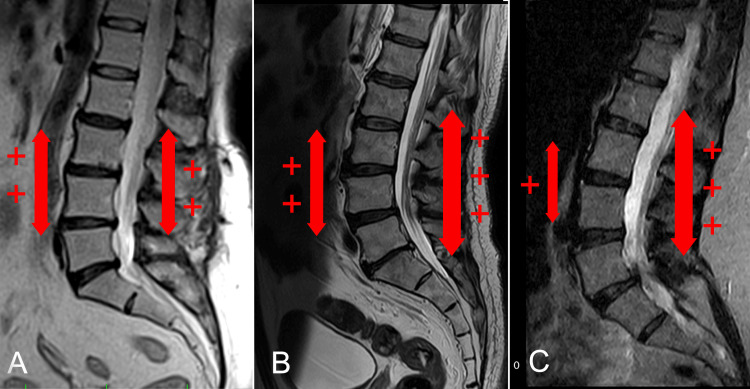
Graphical representation of morphological changes in the sagittal plane of the lumbar spine based on anterior and posterior myofascial tightness A: Balance shifted due to a relative increase in anterior tightness; B: Physiological tension balance; C: Balance shifted due to a relative increase in posterior tightness Image credit: Author Saverio Colonna

The maintenance of physiological spinal curvatures requires a specific balance of tension within the fascial system (Figure 2B). Disruption of this prearranged tension balance may result in alterations to spinal alignment, which can negatively affect biomechanics over time and contribute to the development of lumbar pathology. In the lumbar spine, a reduced tension in the posterior fascial component, relative to the prearranged balance with the anterior component, may have underlying alterations leading to hyper lordosis (Figure 2C). Conversely, excessive tension in the anterior fascial component, relative to the expected posterior balance, may contribute to alterations resulting in hypolordosis (Figure 2A).

Laxity in spinal tissues and reduced resistance to flexion moments have, in fact, been associated with spinal instability [48,49], which may contribute to the onset of LBP [49,50]. When a flexed posture is maintained, passive spinal tissues, due to their viscoelastic properties, gradually undergo deformation, a process known as “creep” [51,52]. This creep deformation increases laxity in passive spinal tissues and decreases resistance to anterior flexion moments [48,50,51,53], thereby initiating a vicious cycle. These mechanical changes are thought to elevate the risk of lower back injuries. Creep in posterior spinal tissues caused by prolonged static flexion not only increases the risk of spinal instability but also leads to heightened activity in the lumbar extensor muscles during the flexion and return phases, triggering lumbar re-extension [48,51]. Increased laxity in passive viscoelastic tissues following prolonged static flexion results in changes in the passively generated extension moment at a given flexion angle. As a result, the lumbar extensors must generate additional force to compensate for the loss of passive tissue stiffness.

It is important to note that the heightened level of muscular activity may be attributed to hyperexcitable responses in the lumbar extensor muscles. Granata et al. [54] demonstrated a progressive increase in the reflex activity of human paraspinal muscles following a period of static flexion-relaxation loading. Similar findings have been observed in animal studies, where passive stretching of ligaments resulted in hyperexcitable reflex responses in the paraspinal muscles [55]. It has been suggested that this heightened reflex activity serves to provide additional spinal stiffness and stability, thereby protecting elongated and compromised ligaments from further injury [54,55]. Recovery of the normal function of viscoelastic tissues begins once the external load is removed, although full recovery may take longer than the duration of the activity that caused the initial alteration [55,56]. Repetitive, prolonged flexion without sufficient recovery time may result in the cumulative creep of lumbar myofascial structures and inflammation of ligaments [55,57], ultimately making the lumbar spine more vulnerable to acute or chronic musculoskeletal disorders.

**Figure 3:**
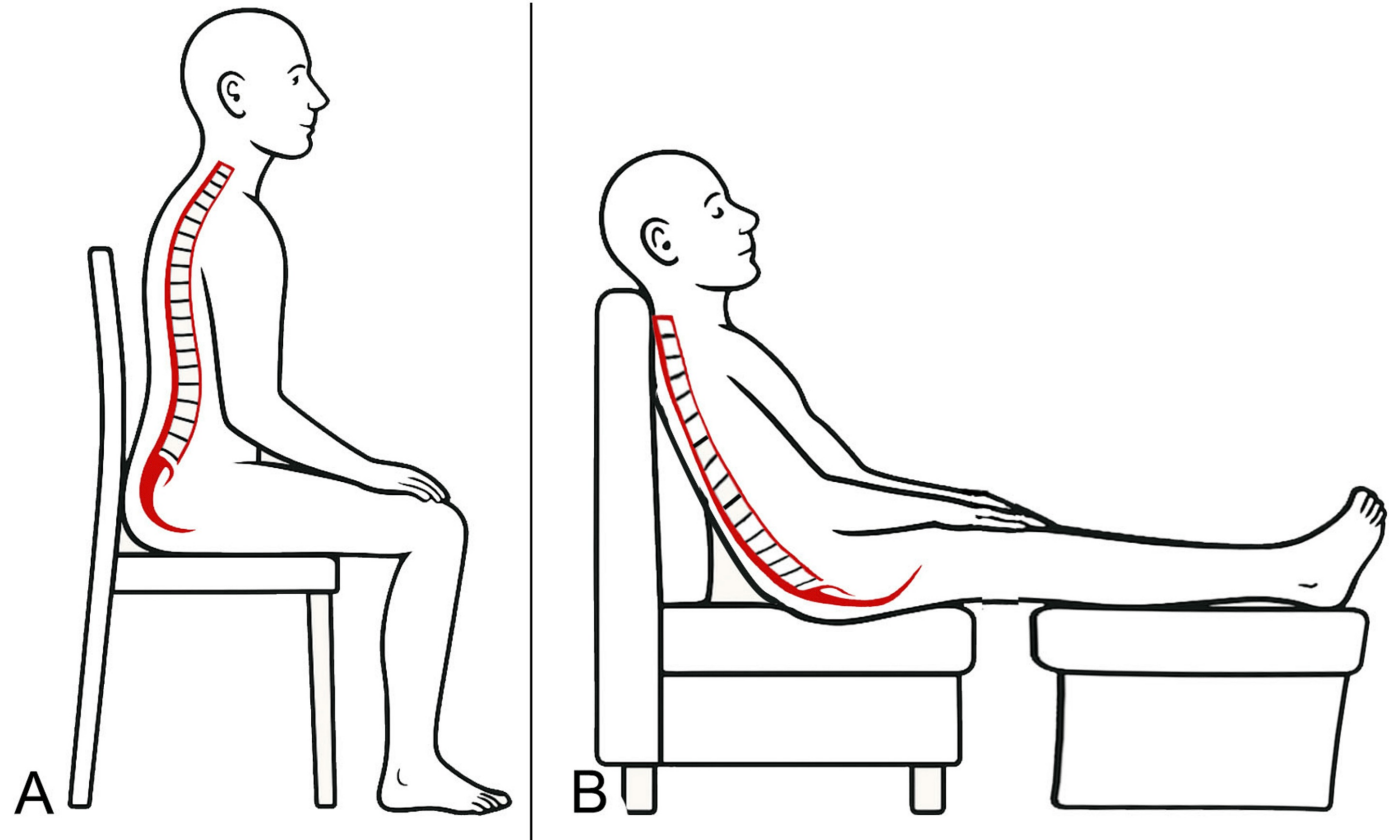
Diagram illustrating the adaptation of the pelvis and lumbar spine in two different sitting postures A: Balanced on the ischial tuberosities; B: Supported by the backrest, with posterior pelvic tilt due to the action of the hamstring muscles and flattening of the lumbar lordosis Image credit: Author Saverio Colonna

In a previous publication [43], we introduced a clinical model in which therapeutic exercise is not merely seen as a tool for muscular strengthening, but as a means of promoting increased stiffness and tension within the fascial system associated with the vertebral complex. It is essential to recognize that overload during standing trunk flexion is not the only cause of myofascial structural creep. During sitting-especially when the position is poorly maintained, the pelvis tends to rotate posteriorly, reducing lumbar lordosis [58]. This risk is exacerbated by prolonged sitting [59,60], particularly due to hip flexion more than knee flexion [61]. It is well known that a slouched sitting posture with increased spinal flexion significantly raises intervertebral disc pressure [62], while also increasing stress on the posterior fascial structures of the spine (Figure 3) [63]. Although increased spinal loading due to poor posture is considered a contributing factor in chronic LBP, its exact etiology remains relatively unclear [3].

Spinal stability

Before delving into a new interpretive model regarding the execution modality of therapeutic exercise, particularly the SBE, it is necessary to further explore certain physiological phenomena related to the myofascial stabilization function of the spine. In 1992, Panjabi proposed a model in which spinal stability depends on the highly coordinated and optimized interactions between three subsystems: the passive subsystem (ligaments, discs, fascia, and bones), the active subsystem (muscles and tendons), and the neural control system [64]. According to this theory, dysfunction in one specific subsystem may be compensated for by adaptive responses in the other two [64,65]. Panjabi also suggested that abnormally increased muscular activation could serve as a stabilizing mechanism to offset spinal instability caused by deficits in other subsystems. This theory has been repeatedly supported by subsequent research [65].

**Figure 4:**
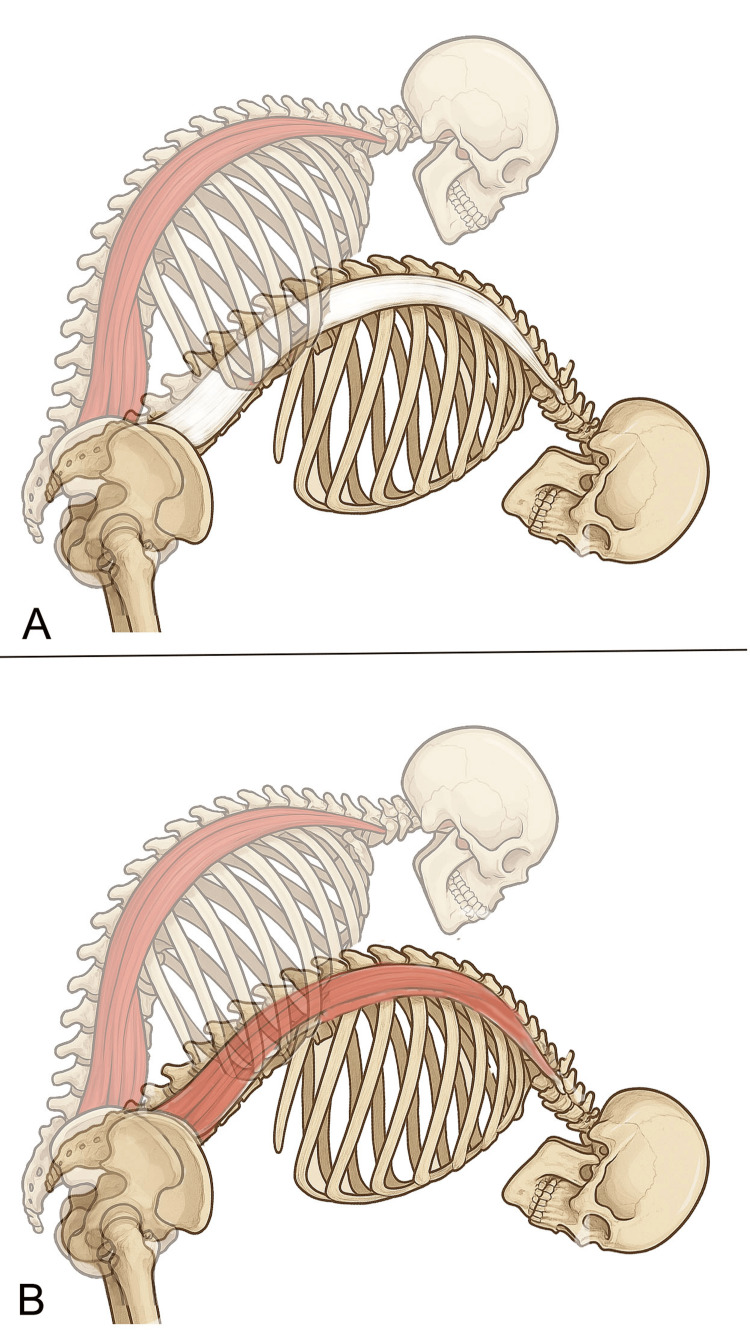
Schematic representation of the flexion-relaxation phenomenon (FRP) during trunk flexion A: Physiological pattern showing cessation of paraspinal muscle activity (indicated by the white erector muscles area) at maximum flexion; B: Non-physiological pattern with persistent paraspinal muscle activation at maximum flexion (indicated by the red erector muscles area) at maximum flexion Image credit: Author Saverio Colonna

To deepen the understanding of spinal stability provided by either passive fascial components or active muscular contraction, examining a neurological reflex known as the flexion-relaxation phenomenon (FRP) is essential. The FRP is defined as the myoelectric silencing of the erector spinae (ES) muscles, recorded through electromyography (EMG), during full trunk flexion in standing posture among asymptomatic individuals (Figure 4A) [66,67]. This phenomenon represents a complete load transfer from active to passive lumbar tissues. In the fully flexed standing posture, the weight of the upper body (trunk, upper limbs, and head) is primarily supported by a passive extension moment generated by the spinal ligaments, intervertebral discs, and the passive fascial components of the extensor muscles [68]. Patients with LBP often exhibit laxity in passive structures and an altered neuromuscular activation pattern in the back muscles, characterized by the absence of FRP in the ES muscles (Figure 4B). Floyd and Silver [66] were the first to describe the absence of ES relaxation during trunk flexion in standing posture among patients with chronic LBP. This finding was later confirmed by more recent studies [69,70], which also included individuals suffering from radiculopathies [70].

The FRP, which occurs during full trunk flexion in standing posture, has also been observed in asymptomatic individuals during slumped sitting [66,71,72]. During sitting, when the lumbar spine approaches full trunk flexion, the transition of load from active (muscular) to passive (ligamentous, fascial, discal) tissues has significant implications for spinal loading [68,72]. Since these passive tissues are viscoelastic in nature, they exhibit both creep and stress-relaxation responses to prolonged tensile loading [56,73]. Several studies [56,73,74] have investigated these creep and/or stress-relaxation responses during “maximum” trunk flexion postures, demonstrating that such mechanical changes lead to an increase in lumbar flexion angle and in the EMG off angle of the lumbar spine, i.e., the angle at which lumbar extensor muscle activity becomes indistinguishable from that observed in full flexion [75]. Previous studies [73] have suggested that, following passive tissue creep, greater force generation by the lumbar extensor muscles is required to compensate for the loss of passive stiffness, indicating a biomechanical compensatory mechanism aimed at maintaining spinal stability.

Appropriate trunk muscle contractions are therefore necessary to execute trunk flexion or to maintain an upright sitting posture and physiological lumbar lordosis [76]. Trunk muscles are traditionally classified into two groups based on their anatomical position [77]: (1) local or deep muscles and (2) global or superficial muscles [78]. Functionally, global muscles contribute to gross trunk movements, whereas local muscles primarily support spinal stabilization [79]. The first group includes deep muscles such as the lumbar multifidus (MF), transversus abdominis (TrA), and internal oblique (IO), which insert directly onto lumbar vertebrae and provide segmental spinal stability [79]. The second group comprises larger and more superficial muscles such as the rectus abdominis (RA), external oblique (EO), and ES, which do not have direct segmental insertions on the lumbar vertebrae [79]. These muscles are involved in gross trunk movement and general trunk stabilization [79].

However, biomechanical analyses by Cholewicki and McGill [80,81] suggest that neither local nor global muscles have a dominant role in lumbar spine stabilization. Similarly, evidence regarding FRP supports the involvement of both the ES and MF during full trunk flexion in both standing and sitting postures, although some studies suggest a more prominent role for the MF [82,83]. It is widely accepted that co-contraction of the deep spinal stabilizers-particularly the TrA, IO, and MF, enhances segmental lumbar and sacroiliac joint stability [84]. Spinal stability has also been associated with trunk stiffness [85], since the elastic stiffness of trunk musculature is considered the primary stabilizing mechanism of the spine [80,86].

The role of lumbar myofascial component in the treatment of LBP

Rehabilitation professionals typically provide structured interventions aimed at restoring normal movement patterns and motor control through a variety of therapeutic approaches. Once a satisfactory level of neuromuscular control is achieved, patients can progress to more demanding exercises. It is crucial that rehabilitation specialists understand the level of core muscle activation across different exercise conditions to determine appropriate progression in exercise prescription and treatment. The comparison between electromyographic muscle activation during various exercises and the maximum voluntary isometric contraction (MVIC) has been used to measure muscle engagement during specific movements [87]. The percentage of activation relative to a muscle’s MVIC during a given exercise reflects the relative difficulty or demand placed on that muscle.

Various types of lumbar muscle strengthening exercises are prescribed to patients with LBP. Among the most common are Pilates exercises, sling-based exercises, and lumbar stabilization routines [88]. When designing a therapeutic exercise protocol for LBP, it is essential to clearly define the intended objectives. Patients with LBP often present with more than a true muscular weakness, with passive tissue laxity and altered neuromuscular activation patterns in the lumbar musculature [89]. This is evidenced by the fact that the FRP of the ES muscles tends to be reduced but not absent in individuals with LBP, indicating greater muscular activation during typical daily movements such as trunk flexion [89]. Consequently, the FRP of the ES muscles has been used to assess LBP and to monitor treatment-related changes over time [90].

However, both asymptomatic and symptomatic individuals may demonstrate asymmetric lumbar flexion via combined lumbar flexion, lateral bending, and axial rotation movements [89]. While most healthy individuals do not report LBP, they may nonetheless exhibit asymmetric FRP responses in the ES muscles due to repetitive movement patterns and poor posture in occupational or daily life contexts [89]. Ning et al. [91] reported that asymmetric lumbar flexion can cause a loss of FRP in the ipsilateral ES muscle even in asymptomatic individuals. Although 90% of subjects without LBP demonstrate an FRP [92], many of them may still be at risk of developing LBP due to asymmetric FRP responses in the ES muscles [92].

Marshall and Murphy et al. [93] reported that a 12-week exercise program including side bridge, supine bridge, partial curl-up, bird-dog, push-ups on a Swiss ball, single-leg hold, and rollout exercises reduced pain perception and ES muscle activity during the relaxation phase of FRP testing in patients with chronic non-specific LBP. According to the authors [93], lumbar stabilization exercises help restore the physiological FRP by strengthening the ES muscle. The change in FRP accounted for 38% of the improvement in Oswestry Disability Index scores after 12 weeks of training. Additionally, the same exercises may lead to re-symmetrization of the FRP in individuals without LBP but with asymmetric FRP patterns in the ES muscles. The aforementioned study establishes a connection between the reduction of LBP symptoms and altered FRP, emphasizing the role of increased strength and/or symmetrical function of the ES muscle. However, as we have previously discussed [43], the abnormal activation of the lumbar paraspinal muscles during full trunk flexion in individuals with LBP appears to be more closely related to reduced capacity of the passive systems located in parallel with the muscle’s contractile elements rather than a dysfunction in the contractile portion itself. Therefore, rehabilitative exercise should be directed more toward enhancing the passive fascial components rather than focusing exclusively on strengthening the active muscular elements [43].

Hamstrings and LBP

The literature has indicated a direct correlation between hamstring tightness and the severity of LBP [94-96]. Increased tightness in the hamstring muscles tends to induce knee flexion [97,98], hip extension, and posterior pelvic tilt [99]. Posterior pelvic tilt is associated with a flattening of the lumbar spine (hypolordosis) [100,101], which in turn increases the risk of LBP [24]. This may be due to increased loading on the anterior spinal structures, including the intervertebral discs [102]. However, some authors [103] argue that pelvic retroversion is a consequence rather than a cause of lumbar lordosis reduction.

The impact of hamstring tightness on lumbar lordosis appears to be more pronounced during trunk flexion than in upright standing posture. López-Miñarro et al. [104] found that hamstring stretching increases anterior pelvic tilt and spinal flexion during seated and supine postures, although they observed no significant differences in standing posture. Li et al. [101] reported that, following a hamstring stretching protocol, there was increased hip contribution during forward trunk flexion, though this did not translate into a significant change in lumbar spine movement. This finding is logical: excessive hamstring tightness may limit pelvic anteversion, which is then compensated for by increased lumbar flexion. This, in turn, may elicit a viscoelastic (creep) response in the fascial structures of the spine, resulting in reduced tightness. Such tightness loss may underlie diminished lumbar lordosis [43], facilitating excessive flexion of the lumbar vertebrae, thereby increasing anterior spine pressure-including on the intervertebral discs.

However, once this dysfunction has been established, regaining physiological hamstring flexibility may not automatically restore normal lumbar movement patterns. For this reason, hamstring stretching is a commonly prescribed therapeutic intervention [94,105], often in conjunction with other treatments, particularly since individuals with LBP tend to exhibit a higher incidence of hamstring tightness [96,106,107]. Despite numerous studies examining the relationship between hamstring flexibility and LBP, the nature of this association is not yet fully understood. This uncertainty is partly due to the inherent difficulties in accurately measuring lumbar spine motion in a motion capture laboratory, as small variations in marker placement, tissue interface, and distribution can introduce substantial variability [108].

Referring back to the previously proposed classification of movement impairment subgroups in LBP [38], we suggested [109] that the flexion-based dysfunction model may be etiologically linked to hamstring shortening. Such altered tightness, by limiting pelvic mobility, imposes greater demands on the lumbar spine. Increased lumbar flexion, in turn, amplifies mechanical stress on the posterior fascial systems [110,111]. When prolonged over time, this stress leads to fascial creep [112]. During trunk flexion, such creep promotes excessive lumbar spine participation at the expense of pelvic movement. This creates a vicious cycle that perpetuates and exacerbates the condition [38].

Tightness of the posterior lumbar muscles and LBP

In patients with flexion-based LBP syndromes, optimal therapeutic exercise should be directed at reducing tightness in the posterior thigh muscles while simultaneously increasing the tightness of the passive fascial system in the lumbar region [42]. This system is primarily represented by the myofascial structures of the ES and MF muscles [109]. To reduce fascial tightness, such as that found in the posterior thigh, our recent publication [113] presents a re-evaluation of the use of stretching techniques, to which we refer for a more detailed analysis. Conversely, when the goal is to increase fascial tightness, in this case in the lumbar region, to restore a more physiological tension balance, the available literature is quite limited.

In another recent work of ours [43], we discussed how physical exercise may represent, although still poorly explored, the most effective method to increase the tone of the fascial system, known as human resting myofascial tone (HRMT) [114]. During muscular contraction, the deep fascial system increases its stiffness [115]. At first glance, the idea of reduced stiffness in the lumbar paraspinal fascial systems may appear contradictory, as several studies [116,117] using shear wave elastography (SWE) have found greater stiffness in subjects with LBP compared to healthy controls. As discussed in our previous work [113], the answer to this apparent contradiction may lie in the abnormal muscular activation observed in LBP patients. This excessive activation, likely compensatory, increases the tension in the fascial systems arranged in series in an attempt to improve vertebral stability, a mechanism referred to as protective muscle contraction.

Indeed, various studies [118-120] report higher electromyographic activity in LBP patients compared to asymptomatic individuals, with the increased muscle activity contributing to greater tension in the serial fascial systems [113]. Most of the literature on connective tissue stiffness has focused on tendons and the myotendinous junctions [121], rather than on muscle-associated fascial components. However, in vitro studies suggest that tendon and muscle fascia may respond differently to mechanical loads induced by exercise [122]. Nevertheless, few researchers have specifically investigated fascial stiffness in response to exercise [123].

Numerous studies [124-130] have explored exercise protocols that target the activation of the ES and MF muscles. However, it has been suggested that the lumbar extensor muscles are notoriously difficult to condition [131,132]. It has also been proposed that valid tests for lumbar extensor strength or endurance require isolation of the lumbar spine through pelvic fixation [133-135]. Otherwise, due to longer moment arms and relatively larger cross-sectional areas, the hip extensors (e.g., gluteus Maximus (GM) and hamstrings) may contribute more significantly to the torque produced [136].

Effectively activating the lumbar paraspinal muscles selectively, without engaging the lower limb posterior muscles, is not straightforward. According to some authors [137], when performing the Roman chair exercise, an activity aimed at strengthening the lumbar paraspinals, increasing the external load leads to a greater increase in activation of the GM and biceps femoris (BF) than of the lumbar extensors. A similar trend is observed during multiple sets: activation of the GM and BF increases more than that of the lumbar extensors, allowing the exercise to be continued with their contribution [137].

A recent study [138] evaluated changes in muscle tightness in the ES and semitendinosus (ST) using myotonometry during isometric trunk extension performed in the prone position, with pelvic and popliteal stabilization. Unlike the previous study [137], this one found that during low-intensity contraction (30% MVIC), the percentage increase in tightness of the ES, ST, and BF was significantly greater than that of the gastrocnemius, suggesting that the ES is the primary lumbar extensor and the ST is the dominant hip extensor among the muscles examined. However, during moderate-intensity contraction (60% MVIC), the tightness increase in the ST exceeded that of the ES, indicating that the synergistic relationship between lumbar and hip extensors changes with increasing load. Another study [139] evaluated the activation ratio between ES and hamstrings during trunk extension at various angles (0°, 10°, 20°, 30°) performed in a prone position, with the trunk suspended and both pelvis and lower limbs secured to the table. The results were consistent with those of the previous study [138].

The role of exercise and fascial tightness

Therapeutic exercise plays a critical role in restoring the optimal viscoelastic properties of fascial tissues, which are often compromised in individuals with chronic musculoskeletal pain, including LBP. Fascia is a dynamic and responsive connective tissue system that adapts to mechanical stimuli. When appropriately loaded, it remodels and improves its ability to transmit forces and provide structural support. While numerous studies have explored exercise protocols for targeting the lumbar paraspinal muscles [140-142], very few have investigated how different execution modes (e.g., isometric, concentric, eccentric, plyometric) might differentially affect the connective fascial component [43]. To explore how exercise modalities influence either the contractile or fascial component of muscles, given the limited literature on the spinal musculature, we will also consider findings from studies on other muscle groups, acknowledging the risk of generalization, as not all muscles respond similarly to mechanical loading.

Among the various types of exercise used in rehabilitation, eccentric exercise is certainly the most widely studied, particularly for its link to the phenomenon known as delayed onset muscle soreness (DOMS). Although any type of sufficiently intense exercise may cause DOMS, eccentric exercise uniquely provokes muscle stiffness and soreness in individuals who are unaccustomed to such training. Eccentric contractions involve the forced lengthening of an actively contracting muscle. A common example is downhill walking.

According to Musat et al. [143], high-load physical exercise can cause injuries which, depending on their anatomical location, are classified into three groups: myofascial, myotendinous, and muscular injuries. Myofascial injuries involve the fascia, especially at the level of the epimysium and its deep extensions (perimysium and endomysium), and may also affect the connective interfaces between fascia and muscle. Myotendinous injuries concern the proximal and distal tendon insertions of muscles. Pure muscular injuries occur away from the fascia and tendons and involve direct damage to muscle fibers, typically characterized by sarcolemmal and sarcomeric destruction [144].

Accurate localization of tissue damage is important for proper diagnosis and therapeutic management [145]. Several studies [146-149] have investigated changes in myofascial stiffness using shear wave elastography (SWE) in response to acute eccentric exercise. However, findings remain somewhat inconsistent. In general, the literature shows that myofascial stiffness, as measured by SWE, increases following eccentric exercise. Shear modulus has been shown to increase immediately or shortly after eccentric exercise for elbow flexors [147,150-152], elbow extensors [153], hamstrings [149,154], knee extensors [155-157], and the triceps surae [158,159].

Nonetheless, not all findings are in agreement: some studies have reported decreased stiffness following eccentric training [160], warranting further analysis and discussion. For example, SWE assessments of the trapezius muscle after eccentric exercise have shown reduced stiffness [161]. Similar reductions have been observed in other muscle groups subjected to various eccentric protocols [162].

As is evident from these studies, none have yet evaluated spinal musculature. Most investigations of post-exercise stiffness have been conducted immediately or within 48 hours after training [163,164]. Only a few studies have assessed stiffness changes at longer time intervals [147,165,166]. One study [165] evaluated stiffness via SWE two weeks after a downhill walking protocol. Results showed increased stiffness in the vastus lateralis (VL) only within the first 24 hours, while the rectus femoris maintained elevated stiffness for up to 14 days.

Lacourpaille et al. [152] reported increased stiffness in elbow flexor muscles persisting up to 21 days following eccentric exercise. However, these results were attributed not to fascial adaptation but to a disruption in calcium homeostasis due to damaged muscle membranes [167]. The authors suggested that increased stiffness observed after eccentric exercise may reflect rapid adaptation in muscle mechanical properties following exercise-induced injury [168].

It is widely acknowledged that one of the main consequences of eccentric exercise-particularly in untrained individuals is the occurrence of nonspecific “muscle damage,” commonly grouped under the acronym DOMS [169,170]. While DOMS may have multiple causes [171], some authors argue that damage to the connective fascial component is the primary contributor [172,173]. This assertion is supported by elevated urinary levels of hydroxyproline and hydroxylysine [173-175], in addition to creatine kinase [176]. Recent research [177-181] increasingly supports the view that damage from eccentric exercise may be predominantly connective rather than contractile in nature, an idea originally proposed by Abraham in 1977 [173].

Although both muscle fibers and connective tissues are affected during exercise-induced muscle damage, the precise sequence of damage between the endomysium and sarcolemma remains inconclusive in current literature. However, since the endomysium is structurally stiffer than the sarcolemma, increased mechanical tension, particularly from eccentric contractions, would theoretically lead to damage of the connective tissue (endomysium) before rupture of the muscle fiber membrane. This hypothesis aligns with their respective mechanical properties: the more rigid structure is expected to bear the initial load and fail before the more elastic sarcolemma. This sequence may help explain the simultaneous increase in hydroxyproline and creatine kinase observed after eccentric exercise. Further research, particularly time-course studies examining microscopic structural damage, will be necessary to clarify this aspect of muscle injury and the subsequent resilient remodeling process.

This perspective applies not only to peripheral muscles, as extensively studied in the aforementioned literature [177-179], but also to the lumbar spinal muscles [182]. Given the considerations outlined above, a spontaneous question arises: if exercise, particularly eccentric training, induces damage at the level of the muscle's connective-fascial system, can we deduce that the subsequent reparative-adaptive response primarily involves the connective-fascial system as well? And if so, what form does that adaptation take? Several studies report that a subsequent bout of eccentric exercise, performed at a certain time interval from the initial one, results in a reduced DOMS response. This adaptive process, known as the repeated-bout effect (RBE), is characterized by lower elevations of muscle damage markers in the blood following repeated exercise sessions, reduced severity of DOMS, less muscle swelling, diminished changes in echogenicity on B-mode ultrasound and/or MRI imaging, and a faster recovery of muscle strength and range of motion following the second bout of exercise [168,183].

Although a significant protective effect can be observed even after a single eccentric bout [184], a more comprehensive adaptation appears to develop after multiple sessions [185]. The RBE is thought to induce a long-lasting protective effect, which can persist for weeks or even months, although its strength tends to decline over time [186,187]. Stauber et al. [188] developed the idea that eccentric exercise causes damage specifically to the muscle’s connective structures by altering the extracellular matrix (ECM). In their study, five subjects completed 70 maximal eccentric contractions of the elbow flexors. Histological analysis of biopsies taken 48 hours post-exercise revealed significant widening of perimysial and endomysial regions, with several samples showing ECM displacement from the muscle fibers, creating an expanded interstitial space.

Furthermore, both human and animal studies following eccentric contractions have demonstrated increased collagen expression and elevated enzymatic activity related to collagen degradation [189]. The increased collagen turnover, marked by heightened activity of matrix remodeling enzymes, is believed to reflect a coordinated remodeling process, indicative of widespread ECM damage. For example, Mackey et al. [189] reported that 100 eccentric contractions of the knee extensors led to elevated serum levels of matrix metalloproteinases for up to 14 days post-exercise. This increase was later followed by heightened collagen IV staining, 22 days post-intervention.

In another study [190], protein synthesis for both myofibrillar and collagen structures was evaluated at 4.5 and 8.5 hours following concentric and eccentric exercise bouts. Results showed a 47% increase in myofibrillar protein synthesis at 4.5 hours and 67% at 8.5 hours compared to baseline, with eccentric training producing a significantly stronger response. However, collagen protein synthesis increased by approximately 300% at both time points, regardless of exercise type. A separate study [191] reported a higher increase in pro-collagen protein synthesis three hours after eccentric exercise when compared to concentric contractions.

In summary, eccentric exercise, characterized by muscular elongation under tension, has been shown to induce specific adaptations that enhance the structural integrity of muscle tissues, particularly the endomysium [192]. The high mechanical loads involved in eccentric contractions stimulate collagen reinforcement within the connective tissue matrix of the muscle [193]. This process results in increased stiffness and resistance of the endomysium, thereby improving its capacity to withstand mechanical stress. Regular application of such exercise stimuli may promote adaptations that fortify fascial structure and resilience, potentially reducing risk of injury.

The breakdown of compromised fascial fibers and their replacement with stronger ones during the repair process serves to reinforce the structure against future mechanical demands. Additional structural adaptations observed following eccentric training include the serial addition of sarcomeres to existing fibers, documented both directly and indirectly via shifts in the length-tension relationship [194], as well as remodeling of intermediate filaments [189], and overall changes in the mechanical properties of muscle tissue, such as increased stiffness.

Application of the SBE in the management of the myofascial component of LBP

The bridge exercise is a widely used strategy in therapeutic settings due to its capacity to improve coordination of trunk muscle activation and reduce pain, while also strengthening both global and local stabilizer muscles [195]. The rationale behind this new proposal for using the SBE in the management of low back disorders is based on the aforementioned concepts, specifically, the classification of LBP into different dysfunction patterns. For the sake of simplification, we will focus only on flexion- and extension-pattern LBP. The use of the SBE in this context aims primarily to increase the tightness of the myofascial structures rather than enhance muscular strength.

Supine bridge exercise in flexion-pattern LBP

Following the rationale previously outlined, the most appropriate variations of the bridge exercise are those that maximally activate the ES, MF, and GM while minimizing activation of the hamstrings. Among the different bridging variations, the SBE has been particularly noted for its selective recruitment of the lumbar paraspinal muscles [196,197]. The SBE performed with the spine in a neutral position and knees moderately flexed appears to effectively activate the GM and gluteus medius (GMed) while minimizing anterior pelvic tilt and excessive lumbar lordosis [197].

Researchers [87] reported that GM activation when SBEs are performed with a bipodal stance on a stable surface and the spine in a neutral position reaches 27% of MVIC when performed with flexed knees. In contrast, when the spine remains in a neutral position but the knees are extended and both feet are placed on a Swiss ball, the same authors observed GM activation at 20% MVIC [87]. Other authors [5] found that during a bipodal bridge on a stable surface, GMed activation reached 28% MVIC, and GM reached 25% MVIC. In comparison, a single-leg bridge on a stable surface recruited the GMed at 47% and the GM at 40% MVIC. For further details on how different bridge exercise variations engage specific muscles, we refer the reader to the narrative review presented in the first part of this work [9].

Lumbar stabilization exercises, especially the posterior bridge, are among the most effective in strengthening lumbar muscles such as the MF [198]. For this reason, the bridge exercise is highly recommended in cases of flexion-pattern LBP, as it can activate both the ES and MF in isometric, concentric, and eccentric modalities [9], with the aim of stiffening the passive fascial systems running parallel to the lumbar spine [43]. When the SBE is performed with the feet placed close to the glutei, hamstring involvement is reduced [9].

**Figure 5:**
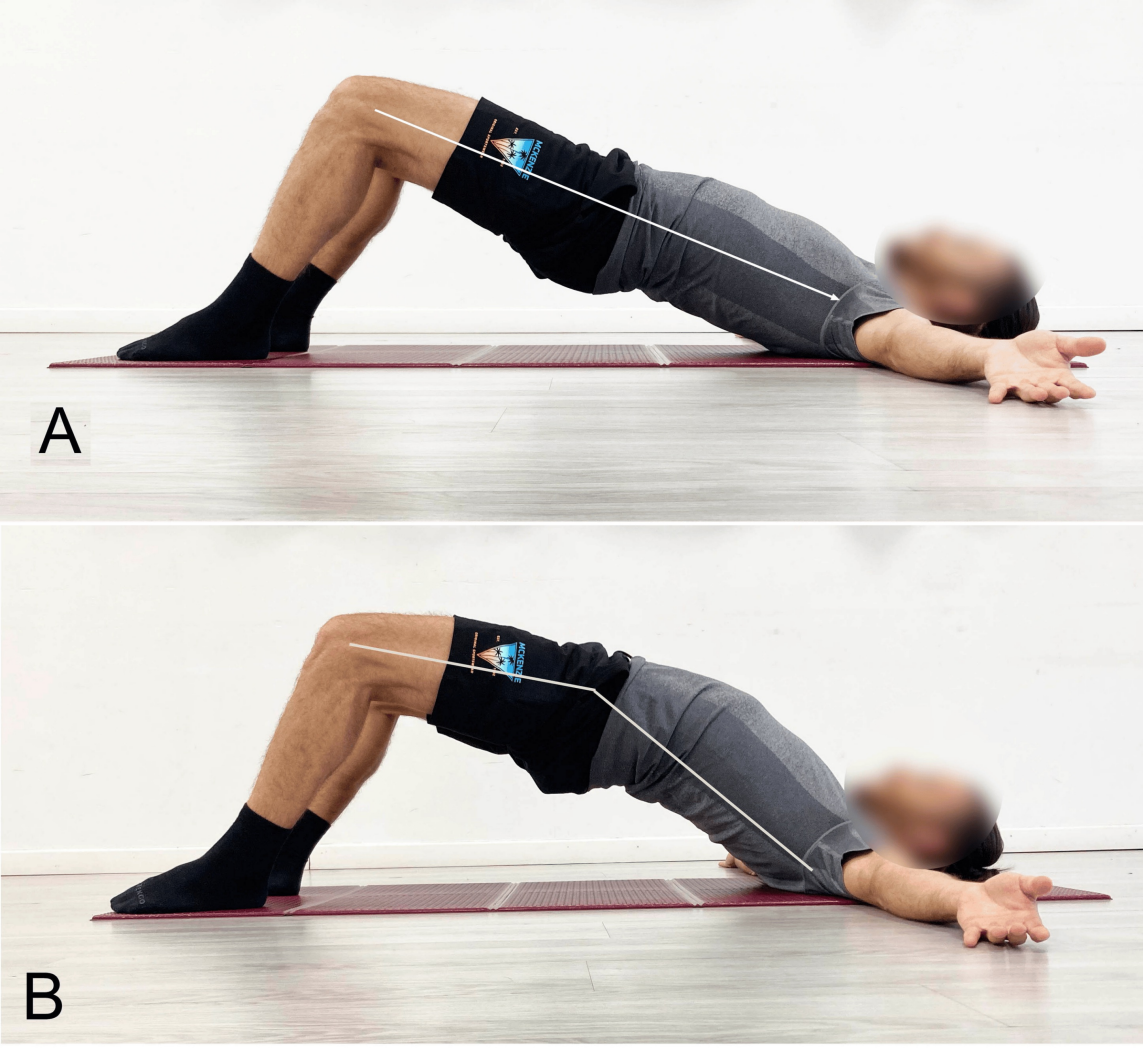
Example of SBE execution A: Neutral lumbar spine; B: Bilateral execution with lumbar hyperextension SBE: Supine bridge exercise Reproduced from Colonna et al. [9], under a Creative Commons Attribution License (CC BY)

In flexion-pattern LBP, contrary to what is generally suggested in the literature [8,199,200], the bridge exercise should preferably be performed in lumbar hyperextension (Figure 5A) rather than in a neutral position (Figure 5B). This is because performing an exercise through a range in which the muscle is relatively lengthened leads to reduced tightness [201], whereas in cases of flexion-pattern LBP, an increase in tightness would be beneficial. For those interested in a more in-depth understanding of SBE in rehabilitation and/or prevention contexts, we refer to the aforementioned review [9], which outlines the principal execution modalities.

**Figure 6:**
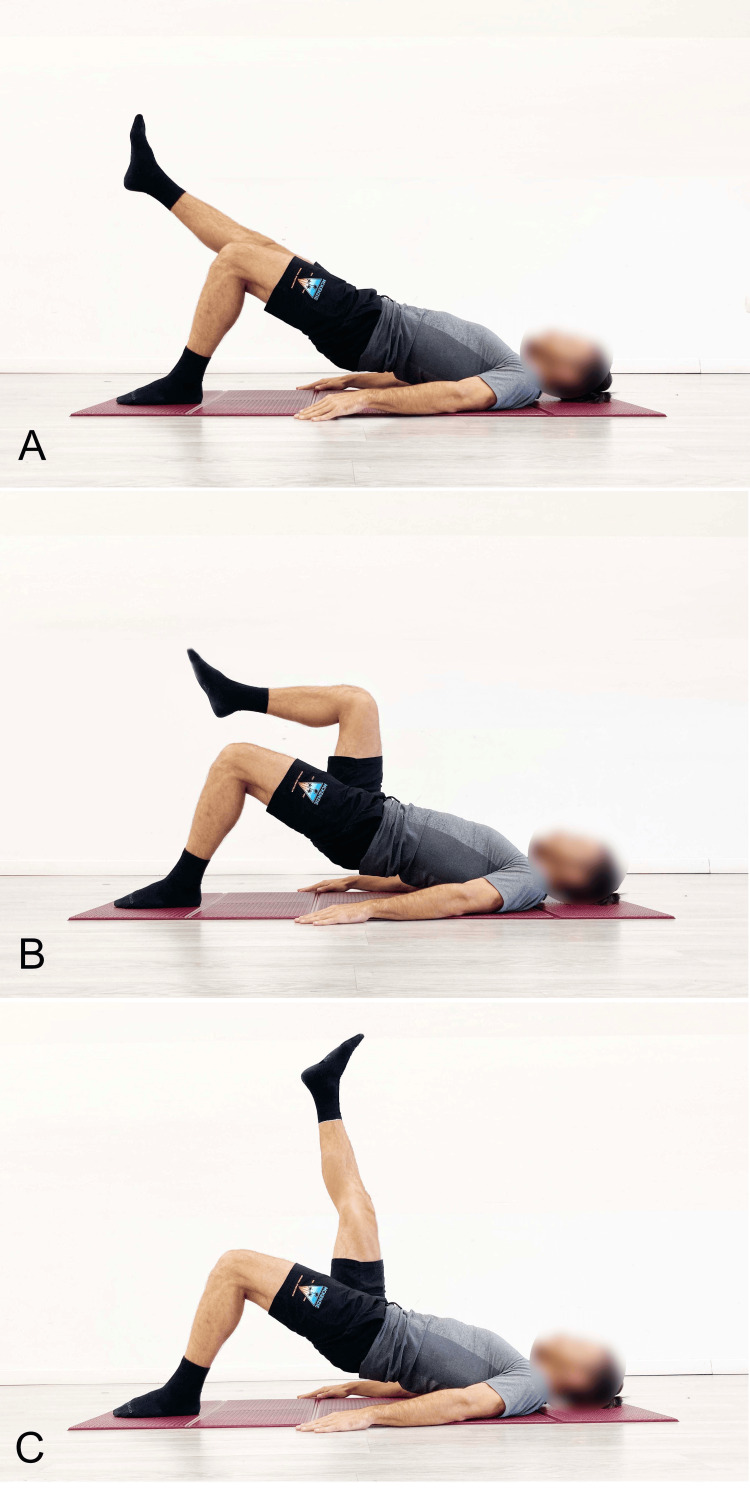
Example of single-leg SBE performed on a stable surface A: The limb suspended in line with the trunk; B: Hip and knee flexion; C: Hip flexion with the knee extended SBE: Supine bridge exercise Reproduced from Colonna et al. [9], under a Creative Commons Attribution License (CC BY)

In our clinical practice, we recommend performing the bridge starting in a bipodal stance (Figure 5) using a concentric contraction to raise the pelvis to the highest possible position, including lumbar hyperextension. The position should then be held isometrically for 20 to 30 seconds, followed by a return to the neutral position via eccentric movement. The set includes four to five repetitions of this cycle. In a therapeutic context, this exercise can be proposed as a tool not only for improving neuromuscular control but also for promoting fascial remodeling by increasing stiffness in the posterior myofascial system of the lumbar spine. This remodeling is thought to contribute to greater spinal stability. To increase the load, the next step involves alternating between bipodal and unipodal execution (Figure 6). From the starting position, the pelvis is lifted in bipodal stance; once the maximum possible extension of the hip and spine is achieved, one leg is raised while attempting to keep the pelvis level.

When performing the single-leg SBE, it is important to be aware that it introduces a predominantly unilateral myofascial load. This selective loading can be beneficial in cases of LBP where tensional dysfunction is present on one side. Additionally, this type of exercise also imposes a torsional load [9]. Torsional loads can be particularly useful in the treatment of flexion-rotation syndromes, although they are more complex to manage. The narrative review on the SBE also presented the different modalities of performing the single-leg version [9]. The duration of the isometric hold depends on the subject's level of conditioning: one may start with five to 10 second holds and progress to 20 or 30 seconds.

**Figure 7:**
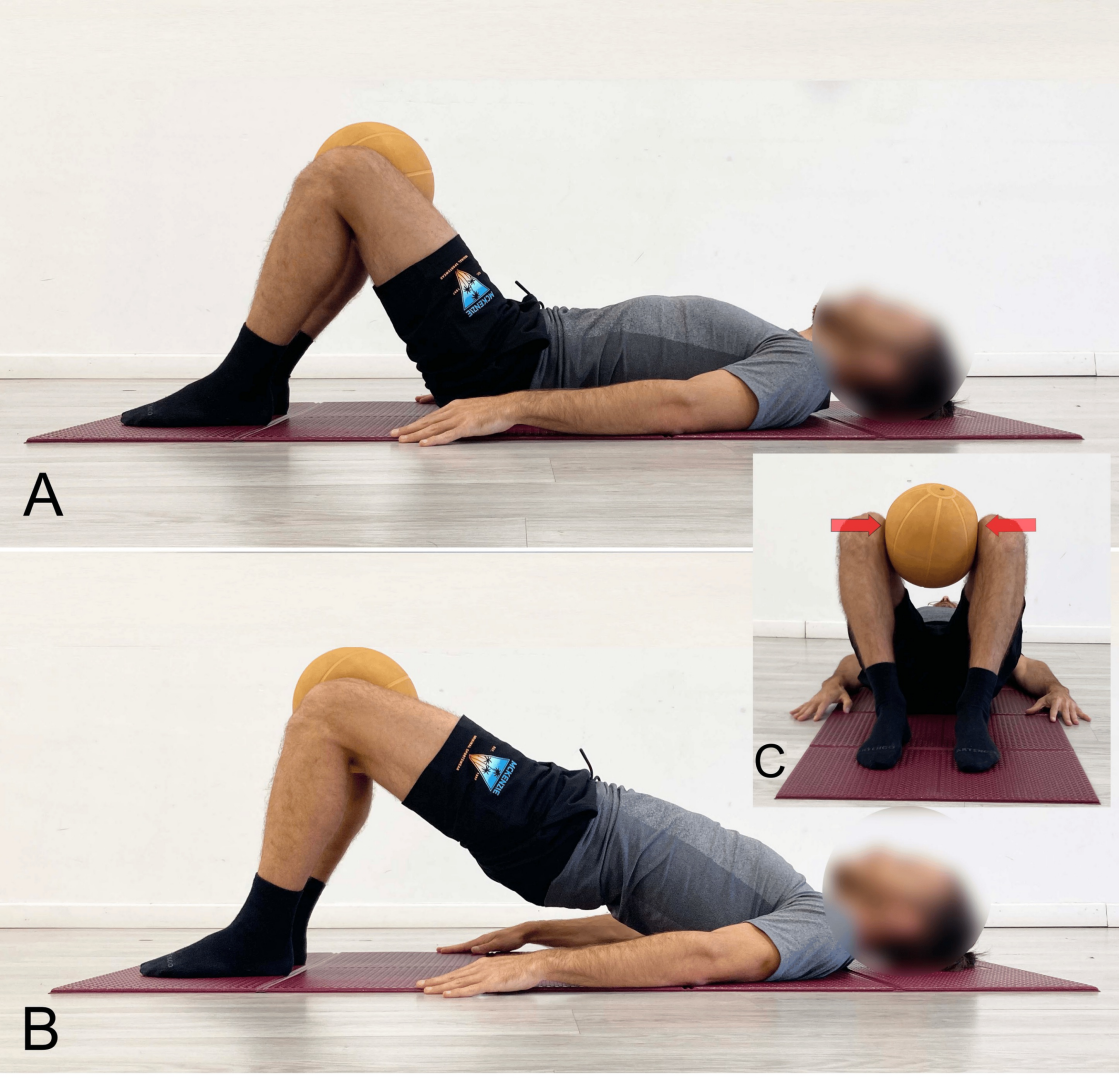
Example of SBE with different knee flexion angles performed on a stable surface A: 60° starting position; B: 60° end position; C: 90° starting position; D: 90° end position; E: 120° starting position; F: 120° end position SBE: Supine bridge exercise Reproduced from Colonna et al. [9], under a Creative Commons Attribution License (CC BY)

In flexion-pattern syndromes, since the posterior spiral myofascial chains are typically over-recruited [109], i.e., chains which include the hip abductor and external rotator muscles, it is advisable to also activate the hip adductors during the classic SBE by squeezing a ball placed between the knees (Figure 7) [9]. This activation, due to the reciprocal inhibition reflex [202], may inhibit the contraction and facilitate the lengthening of the myofascial components of the antagonist muscles, namely the abductors.

Supine bridge exercise in extension-pattern LBP

**Figure 8:**
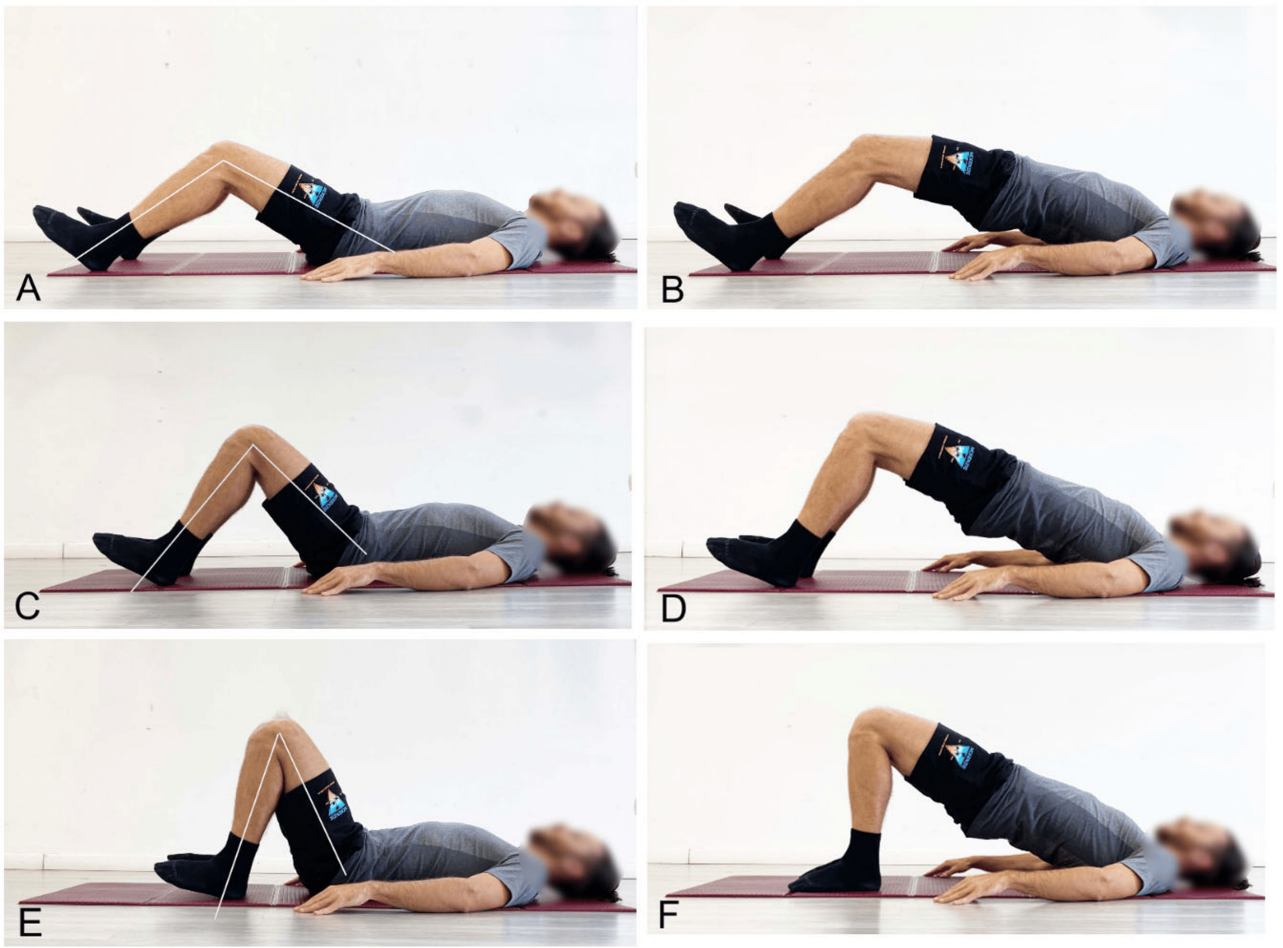
Example of SBE with different knee flexion angles performed on a stable surface A: 60° starting position; B: 60° end position; C: 90° starting position; D: 90° end position; E: 120° starting position; F: 120° end position SBE: Supine bridge exercise Reproduced from Colonna et al. [9], under a Creative Commons Attribution License (CC BY)

**Figure 9:**
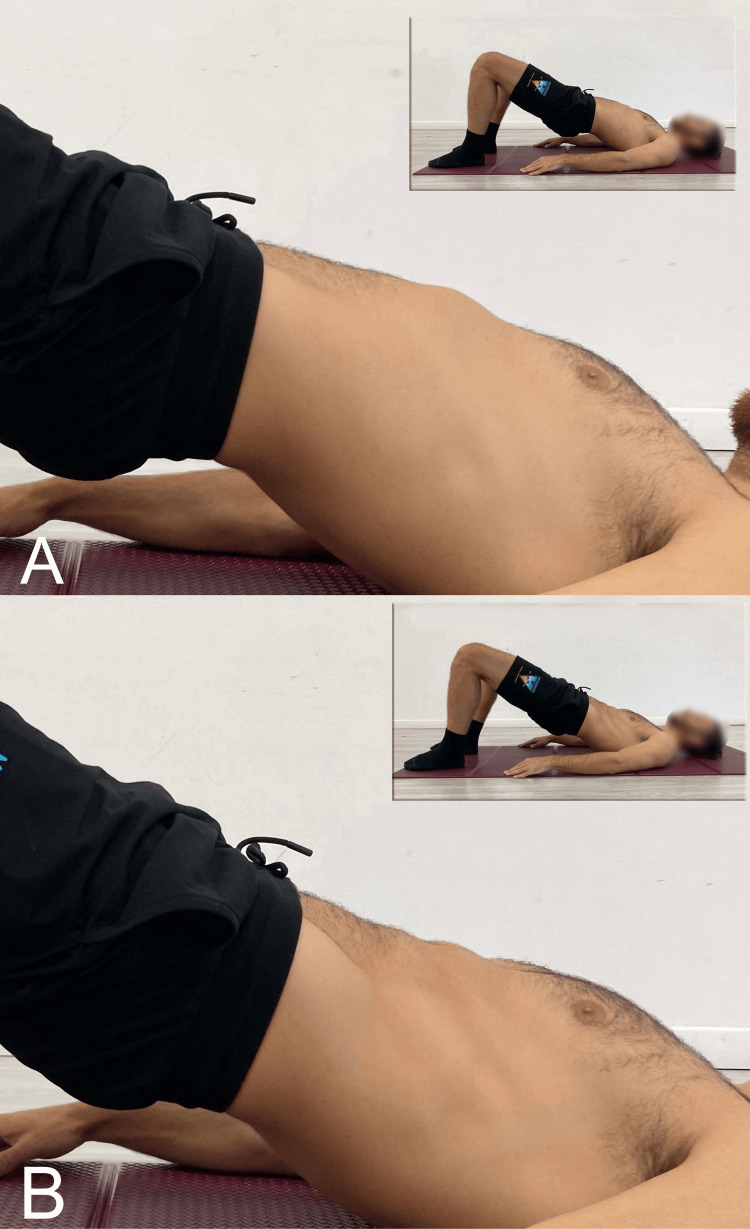
Execution of SBE A: Traditional; B: With the abdominal drawing-in maneuver SBE: Supine bridge exercise Reproduced from Colonna et al. [9], under a Creative Commons Attribution License (CC BY)

**Figure 10:**
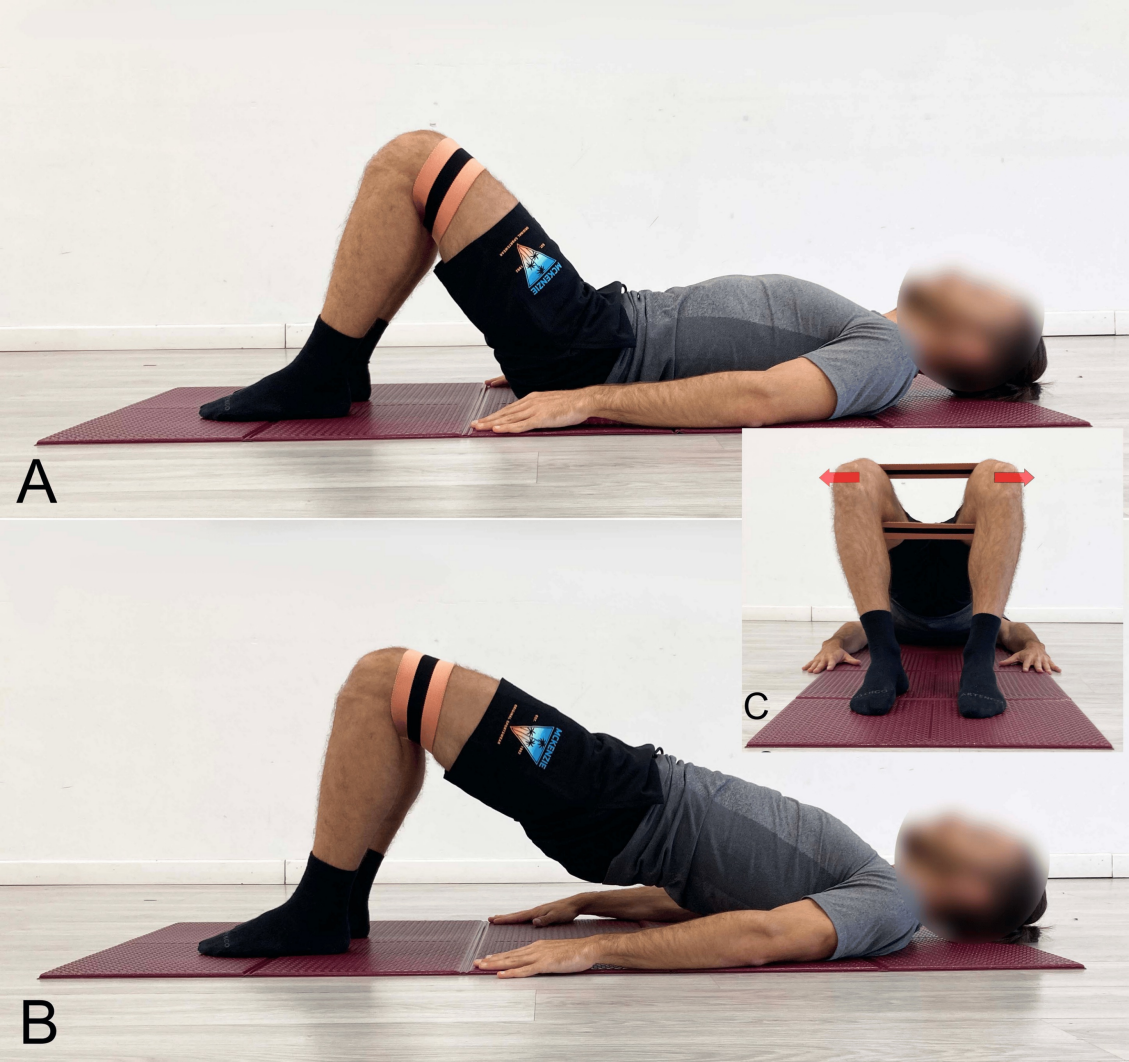
Bridging exercise with added activation of the abductor muscles using an elastic band A: Starting position; B: End position, lateral view; C: End position, foot-level view Reproduced from Colonna et al. [9], under a Creative Commons Attribution License (CC BY)

**Figure 11:**
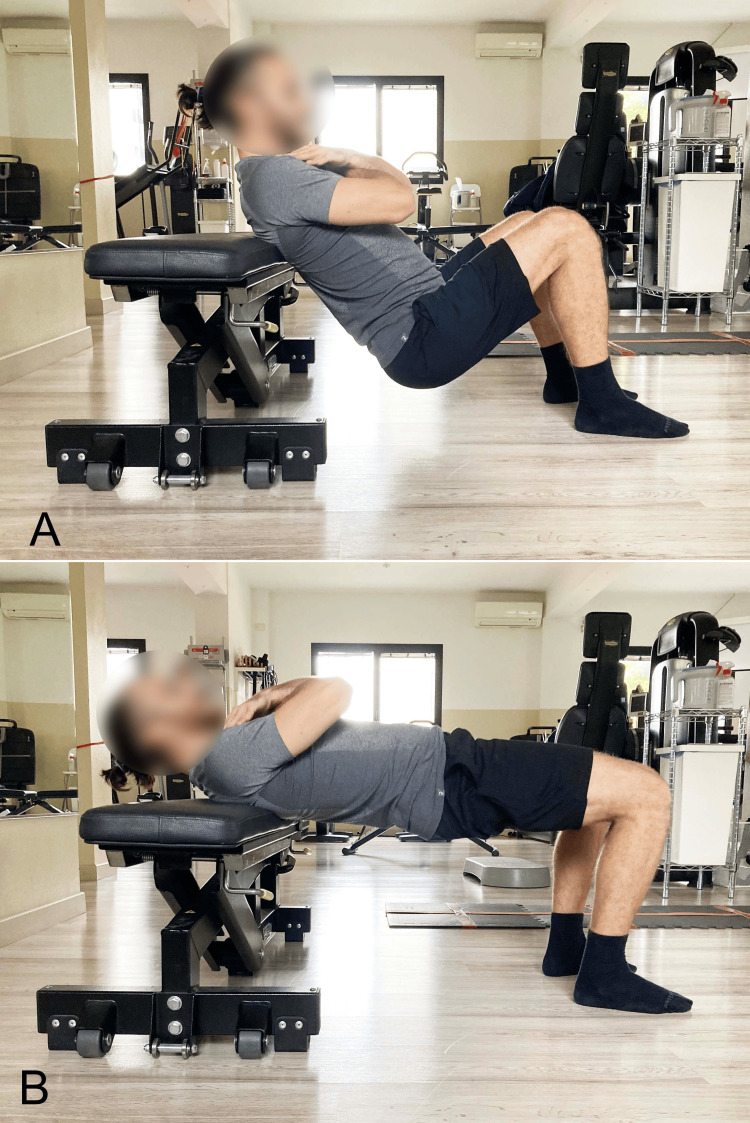
Barbell hip thrust with dorsal support on a bench A: Starting position; B: End position. Reproduced from Colonna et al. [9], under a Creative Commons Attribution License (CC BY)

In the case of extension-pattern LBP, too, the SBE is recommended but with specific precautions. Pelvic elevation should primarily result from hip extension, while the spine must not exceed a neutral position (Figure 5) to reduce tightness in the hip flexor myofascial systems. Knee flexion between 60° and 90° is advisable to engage the hamstrings, whose length is inversely proportional to lumbar lordosis (Figure 8) [203]. To increase tightness in the hip extensor myofascial systems to further limit lumbar extension during the exercise, the simultaneous contraction of the abdominal muscles (abdominal drawing-in maneuver) is recommended (Figure 9) [204]. Concurrent activation of the hip abductor muscles should be incorporated using a resistance band placed around the knees (Figure 10) [9], which, via reciprocal inhibition, may promote myofascial relaxation of the adductor-hip flexor muscles that are typically over-recruited in hyperlordosis [42]. To further reduce the likelihood of working in lumbar hyperextension, we recommend performing the SBE in the barbell modality (Figure 11), as this variation appears to facilitate limiting lumbar spine hyperextension.

Activation of hip extensors, such as the hamstrings and GM, should induce posterior pelvic tilt, which is normally associated with a reduction in lumbar lordosis. This activation should also concurrently inhibit the myofascial activity of the hip flexors, which tend to be shortened in hyperlordosis [205]. We recommend performing the bridge starting in a bipodal stance using a concentric contraction to raise the pelvis to the highest possible position, avoiding lumbar hyperextension. The position should then be held isometrically for 20 to 30 seconds, followed by a return to the neutral position via eccentric movement. The set includes four to five repetitions of this cycle. By selectively modifying the execution of the SBE based on the patient's functional diagnosis-particularly the underlying movement impairment syndrome, we can maximize the therapeutic effects on both the muscular and fascial systems of the lumbar spine.

Limitations and future directions

The proposed rationale for the SBE as a myofascial intervention for LBP is based on biomechanical models, clinical observations, and available literature on exercise prescription and spinal stability. However, several limitations must be acknowledged. First, although the fascial interpretation of the SBE offers an innovative perspective, it is largely theoretical and requires empirical validation. There is currently limited direct evidence demonstrating that SBE modulates fascial stiffness or improves myofascial tension in a quantifiable manner. Future research should include biomechanical and imaging-based studies to assess the acute and chronic effects of the SBE on fascial structures, particularly the thoracolumbar fascia, sacrotuberous ligament, and gluteal aponeuroses. Second, this article draws on data from studies with heterogeneous methodologies and participant populations. While this allows for broader conceptual integration, it also limits the specificity of conclusions drawn. Controlled clinical trials investigating subgroup-specific effects of the SBE, such as differences between flexion-based and extension-based LBP presentations, are needed to refine exercise protocols and validate individualized approaches. Additionally, most current research focuses on the SBE's effects on muscle activation and spinal kinematics, with relatively little emphasis on the fascial system. Developing assessment tools that measure fascial mobility, elasticity, and thickness may allow for more targeted interventions and personalized programming in the future. Finally, although the clinical execution strategies proposed here are grounded in movement science and functional anatomy, their effectiveness remains to be formally tested. Randomized controlled trials and longitudinal outcome studies would be valuable in assessing the real-world impact of this approach on symptom reduction, functional recovery, and recurrence prevention in chronic or recurrent LBP.

## Conclusions

The SBE represents a valuable component within rehabilitation programs targeting LBP, due to its capacity to activate both local and global trunk stabilizers. Beyond traditional interpretations focused on muscle strengthening, the present work introduces a novel clinical model wherein the SBE is employed as a therapeutic strategy aimed at modulating the mechanical properties of the passive myofascial system, particularly increasing fascial stiffness to enhance segmental spinal stability. This second installment of a three-part series builds upon previous biomechanical insights by proposing specific execution protocols tailored for functional subgroups of LBP, as defined by the Movement Impairment Syndromes framework. In flexion-based LBP, emphasis is placed on selectively recruiting the lumbar paraspinal muscles while minimizing hamstring involvement, thereby restoring posterior myofascial tone. In extension-based presentations, the SBE is adapted to reduce lumbar hyperextension, promote posterior pelvic tilt, and facilitate co-contraction of abdominal and hip musculature, contributing to neuromuscular rebalancing. This individualized, diagnosis-driven approach underscores the clinical relevance of matching exercise prescription to the patient’s specific movement impairment pattern. It further supports the integration of fascial considerations into rehabilitation paradigms, with the goal of improving spinal function, reducing symptomatology, and preventing recurrence in patients with chronic or recurrent LBP.
